# The role of calcium and predation on plate morph evolution in the three-spined stickleback (*Gasterosteus aculeatus*)

**DOI:** 10.1002/ece3.1180

**Published:** 2014-09-01

**Authors:** Carl Smith, Rowena Spence, Iain Barber, Mirosław Przybylski, Robert J Wootton

**Affiliations:** 1 School of Biology, University of St. AndrewsSt. Andrews, KY16 8LB, U.K; 2 Department of Biology, University of LeicesterLeicester, LE1 7RH, U.K; 3 Department of Ecology and Vertebrate Zoology, University of ŁódźŁódź, Poland; 4 Institute of Biological, Environmental and Rural Sciences, Aberystwyth UniversityAberystwyth, Ceredigion, SY23 3DA, U.K

**Keywords:** Adaptation, calcium concentration, *Gasterosteus aculeatus*, natural selection, phenotypic adaptation, selective predation

## Abstract

While the genetic basis to plate morph evolution of the three-spined stickleback (*Gasterosteus aculeatus*) is well described, the environmental variables that select for different plate and spine morphs are incompletely understood. Using replicate populations of three-spined sticklebacks on North Uist, Scotland, we previously investigated the role of predation pressure and calcium limitation on the adaptive evolution of stickleback morphology and behavior. While dissolved calcium proved a significant predictor of plate and spine morph, predator abundance did not. Ecol. Evol., xxx, 2014 and xxx performed a comparable analysis to our own to address the same question. They failed to detect a significant effect of dissolved calcium on morphological evolution, but did establish a significant effect of predation; albeit in the opposite direction to their prediction.

## Introduction

We are grateful to MacColl and Aucott (2014) for prompting discussion of our work and for furthering the debate on three-spined stickleback (*Gasterosteus aculeatus*) morphological evolution. Despite several decades of research, it is surprizing that the selective agent for morphological evolution in this species remains a subject of active research. We also agree that it is instructive, and somewhat sobering, that different research groups working on largely the same populations and using comparable data can draw conflicting conclusions. However, as we will show, our conclusions are actually not as divergent to those of MacColl and Aucott (2014) as might appear.

The three-spined stickleback is a valuable model for understanding the mechanism of evolution in nature through the evolution of its bony plates and spines. The size, number, and arrangement of these bony elements show wide variation, although most populations predominantly express a single morph, that is either the *complete* morph, with a continuous row of plates from immediately behind the head to the caudal peduncle; the *partial* morph with an anterior row of plates, then a length of the body that lacks plates, succeeded by a posterior row of plates, and the *low* morph with only an anterior row of plates and the remainder of the body naked. There is a striking correlation between the frequency of these morphs and the ionic concentrations of the water in which they live, for at least part of their life (Heuts 1947; Wootton 1984, 2009). *Complete* and *partial* morphs are associated with marine and estuarine conditions, although some populations migrate into freshwater to spawn in spring. *Low* morphs usually reside in freshwater throughout their life (Wootton 1976). This pattern is not universal, and resident freshwater populations of the *complete* morph have been recorded from eastern Europe, eastern North America, and northeastern Asia (Wootton 1976; Hagen and Moodie 1982; Bańbura 1994).

In a few locations, including the Hebridean Island of North Uist off northwest Scotland, sticklebacks display morphs with plates and spines more reduced even than in the freshwater *low* plate morph. In some populations, fish have no dorsal and pelvic spines, or lack a pelvic girdle altogether. In others, all but a vestige of the lateral plates is present (Campbell 1985). Consequently, the three-spined stickleback populations of North Uist offer an exceptional opportunity to examine the selective agent for plate and spine reduction. In a recent study (Spence et al. 2013), we investigated the role of predation pressure and calcium availability on the evolution of stickleback morphology in 36 populations on North Uist. We concluded that dissolved calcium was a significant predictor of plate and spine morph, while we failed to detect an effect of predator abundance. The chief predator of three-spined sticklebacks on North Uist is believed to be the brown trout (*Salmo trutta*).

MacColl and Aucott (2014) raise three main issues with our work, and we will address each in turn. Our aim is that these responses will contribute to a broader discussion of the likely role of dissolved calcium and predation rate on understanding morphological evolution in sticklebacks.

### Qualitative measure of stickleback plate morph

In Spence et al. (2013), we classed sticklebacks as either belonging to a “normal” freshwater *low* plate morph or a *minimal* morph. The *minimal* morph included discrete, previously described morphs with unusual degrees of plate and spine reduction. These were: *plateless,* with no lateral plates and no reduction in the spines or pelvic girdle; *spine-deficient plated* with thoracic plates, but no dorsal and pelvic spines; and *spine-deficient plateless,* with no lateral plates and reduced or absent dorsal spines, ventral spines, and pelvic girdle (Campbell 1985). We adopted this conservative approach, of grouping highly reduced morphs, to maximize statistical power, but also because it reflected our view that the same agent of selection is likely to select for a reduction in any or all of these skeletal elements, whether selection is generated from a deficiency of dissolved calcium or an absence of predators.

MacColl and Aucott (2014) adopted a different approach, and instead measured seven spine and plate variables and compressed these measurements in a principal component analysis (PCA), using PC1 as a continuous variable of what they termed “armour PC”. All spine and plate variables in their analysis showed strong positive loadings in PC1, which accounted for a high proportion of variance (70%), implying that these variables are all highly correlated (unfortunately, eigen values are not provided for the PCA). A high correlation of variables contributing to PCA is not desirable, as the variables simply mirror each other and do not independently contribute to the PC. Ideally, the variables used in PCA should correlate weakly with each other, but should contribute independently to the variable of interest, in this case what comprises “armouredness”. Thus the parameter “armour PC” demonstrates high internal redundancy and, hence, has limited general validity. A result is that “armour PC” does not provide any particular insights into variation within morph classes. Consequently, it is difficult to see what added information this approach provides over the more robust *low* versus *minimal* classification used in Spence et al. (2013), which unquestionably captures a meaningful distinction between these two morph types. In fact, MacColl and Aucott (2014) do acknowledge as much in their discussion, and their analysis using our binomial classification of morphs generates the same result as their PCA analysis.

### Measurement of dissolved calcium concentration

MacColl and Aucott (2014) adopted a more comprehensive procedure for measuring dissolved calcium than the one used in Spence et al. (2013), and one that undoubtedly generates more precise estimates. Overall, our measurements of dissolved calcium were consistently lower than those of MacColl and Aucott (2014), although not of Giles (1983). While we readily accept that the assay outlined by MacColl and Aucott (2014) is more precise than our own, the basis to MacColl and Aucott’s (2014) criticism of our dissolved calcium measurements hinges on an argument that the LaMotte water quality test kits we used are suitable solely for measuring water quality in swimming pools. This is a disingenuous assertion, as the most cursory inspection of LaMotte’s website and literature clearly indicate that their water quality test kits are intended for a range of purposes, but particularly environmental and waste water monitoring.

The distribution of dissolved calcium concentrations among our 36 fieldsites on North Uist was strongly bimodal. Lochs on North Uist are essentially calcium poor or calcium rich, corresponding with whether they are located on a band of calcium-rich shell-sand grassland, termed the *machair*, that supports rich vegetation and alkaline lochs, or blanket peat bogs supporting highly acidic lochs (Beveridge 2001; Friend 2012). For the subset of our sites that MacColl and Aucott (2014) also measured dissolved calcium concentration, our data and theirs agree in assigning lochs to these two distributions. Thus, while our estimates lack the precision of those of MacColl and Aucott (2014), our respective discrimination of calcium poor and calcium rich sites was identical. We also note that our measurements of dissolved calcium correlate significantly both with those of MacColl and Aucott (2014) and Giles (1983), who measured the same sites 30 years earlier.

Differences between our data and those of MacColl and Aucott (2014) *within* low calcium sites might be owing to our less precise methodology. An additional or alternative explanation for this difference may also relate to the extremely low concentrations of dissolved calcium in the acid lochs, which may be susceptible to variation in response to patterns of rainfall and runoff. We collected fish and water samples between 2010 and 2012. We also undertook a comprehensive resampling of loch water samples in June 2012, to ensure consistency among water samples by minimizing year effects. Thus, 29 of the 36 sites listed in Table 1 of Spence et al. (2013) derived from resampling in June 2012, with data for the remaining 7 sites (which we were unable to revisit in 2012), based on our original samples from 2010. The level of rainfall in June 2010 and 2012, when we collected our water samples, and May 2011 when MacColl and Aucott (2014) collected their water samples, differed markedly. Thus the level of rainfall measured by the UK Met Office at Stornaway airport in May 2011 was 153.2 mm, while in June 2012, it was 37.8 mm, and in June 2010, it was 28.2 mm (Met Office 2014). This means that the level of precipitation differed by >400% between our respective samples. The slightly higher concentrations of dissolved calcium detected by MacColl and Aucott (2014) may be, partially or wholly, a consequence of the effects of heavy rainfall during their collection period, and the consequent runoff of dissolved nutrients carried from the surrounding landscape and into the lochs, resulting in marginal, but significant, changes to water quality parameters. In Spence et al. (2013), we commented that the dissolved calcium concentration at 4 of our sites (Lochs Croghearraidh, Sanndaraigh, Hosta, nan Athan) were conspicuously lower in our samples than those recorded by Giles (1983), although without identifying precipitation as a possible explanation.

### Measurement of predation

In Spence et al. (2013), we used trout abundance based on angler catch returns as a proxy measure of predation rate. Measuring predation rates and specifying the rate of prey consumption by predators in ecological studies are notoriously difficult (Abrams and Ginzburg 2000), and we raised this weakness as a potential caveat to our findings in the article.

In contrast, MacColl and Aucott (2014) used records from angling competitions as a proxy for predation rate. A weakness of these data is that they are from a limited subset of sites. Hence, the analysis in Table 2, in which MacColl and Aucott (2014) correlated stickleback morph with dissolved calcium and angler’s catches, is based on a sample size of just 12, rather than the 36 sites used in our analysis. We also note that MacColl and Aucott’s (2014) estimate of trout abundance, based on the overlapping subset of sites for which trout catch rate data from angling competitions were available, correlate significantly with our own estimates of trout abundance.

The weakness of using catches from angling competitions is that these data are unlikely to come from a random subsample of sites, as competitions would not be staged at lochs supporting only small trout, or where trout densities were low. Data for fish sizes within lochs are also unlikely to represent unbiased subsamples, as anglers typically target the largest fish in a population (MacColl et al. 2012). Thus the analysis in MacColl and Aucott’s (2014) Table 2 is presumably (we are not sure, as they do not name their study sites) those populations with the highest trout abundances and with fishing effort within populations targeted at the largest individuals. A low sample size and likely bias in fish sampling data may explain the significant effect of trout abundance on plate morph presented by MacColl and Aucott (2014), which we did not detect in our analysis. If MacColl and Aucott (2014) were to increase their sample size and include sites with low trout abundance, the pattern of correlation between trout abundance, dissolved calcium, and stickleback morph might change.

A surprising outcome of MacColl and Aucott’s (2014) analysis was that their results indicated an effect of predation on stickleback morph evolution, that is in the opposite direction to their predictions. Thus, their results show that at high predator abundance, sticklebacks evolve reduced “armour”; that is, where there are most predators, sticklebacks possess smaller and fewer plates and spines, or none at all. This outcome contradicts the literature they cite to support their arguments (e.g., Reimchen et al. 2013) and numerous other studies on the impact of predatory fishes (Wootton 1976, 1984; Bańbura et al. 1989; Reimchen 1994, 1995, 2000). This finding may be symptomatic of the flaws, and we have identified in their estimation of predator abundance.

In Spence et al. (2013), we additionally collected behavioral data on sticklebacks from 16 populations that varied in trout abundance, to examine whether predation risk affected fish latency to emerge from a refuge. This is a standard behavioral test of risk-taking, that is known to be highly sensitive to predation risk (Brown and Braithwaite 2004). We detected a significant effect of trout abundance in populations with *minimal* plates and spines, with populations exposed to a high abundance of predators significantly less bold. These data provide additional, independent evidence that our assessment of predator abundance accurately reflected predation risk, and in the predicted direction.

## Discussion

Four possibilities present themselves with respect to plate and spine morph evolution in the three-spined stickleback. The first is that dissolved calcium is the primary agent of selection. An alternative is that predation plays the major role. A third possibility is that dissolved calcium and predation interact, possibly in subtle and complex ways to drive plate and spine morph evolution. A final possibility is that neither dissolved calcium or predation play a significant role, and another agent of selection is responsible.

The correlation between dissolved calcium and stickleback plate morph is so striking that it is impossible to overlook. At all sites in North Uist, three-spined sticklebacks with reduced plates and spines are *only* found in lochs with low dissolved calcium concentrations, irrespective of trout abundance. If predation were the sole variable driving morph evolution, highly reduced morphs (what we term *minimal* morphs) would be predicted in some of the calcium-rich *machair* lochs with few predators, but they are never found in those sites. This pattern of morphology reflects the more widespread step-change between the distribution of plate morphs in marine and freshwater populations. Thus, *complete* and *partial* populations are found almost exclusively in the marine environment, while *low* morph fish are exclusively found only in freshwater. Imposed on this general and almost universal pattern are some rare exceptions that challenge our understanding of the evolution of stickleback plate morphs. Thus resident freshwater populations of the *complete* morph are described from central and eastern Europe, eastern North America, and northeastern Asia (Wootton 1976; Hagen and Moodie 1982; Paepke 1983; Bańbura 1994). Notably, those regions in which the *complete* morph occurs in freshwater all experience extremely low winter temperatures (Wootton 1976). There is also evidence that the mechanisms for calcium and osmotic regulation in these populations may diverge from that of populations displaying the more typical distribution of plate morphs (Spence et al. 2012). However, our understanding of why *complete* populations of sticklebacks are sometimes resident in freshwater remains incomplete.

Support for a role of dissolved calcium availability in stickleback morphological evolution is primarily correlational. However, the question of whether dissolved calcium can impose selection on stickleback skeletal growth can be tested experimentally, and just such an experiment was conducted by us (Spence et al. 2012). We measured the independent effects of dissolved calcium and salinity on the growth rate of sticklebacks and detected a significant interaction of both with plate morph. Stickleback morphs with the most extensive plate and spine development showed significantly lower growth rates when exposed to low compared with high dissolved calcium concentrations, while sticklebacks with limited plate and spine development experienced no impact of calcium concentration on growth. These findings strongly implicate a role for calcium as a limiting element in skeletal growth. Low dissolved calcium concentrations used in Spence et al. (2012) matched the concentration of acid lochs on North Uist.

Interestingly, MacColl et al. (2012) observed that the growth rates of sticklebacks in acid lochs on North Uist are unusually slow, with fish achieving a maximum size considerably smaller than any other populations for which data are available. This pattern is not seen in lochs on North Uist with high dissolved calcium concentrations. The slow growth and small body size of sticklebacks from acid lochs might be a consequence of poor feeding conditions. However, this effect might also be a result of growth limitation imposed by low calcium availability. If the case, a prediction is that natural selection will impose constraints on investment in skeletal structures that are not critical to development in calcium-poor environments. The stickleback external skeleton, comprising plates and spines, is potentially a target for this selection (Spence et al. 2012). Thus, the various *minimal* morphs on North Uist may represent the outcome of selection to economize in the allocation of calcium to skeletal growth.

Trout are important predators of sticklebacks, in North Uist and elsewhere (Wootton 1976, 1984; Reimchen 1994, 1995, 2000). Thus our finding that predation had no significant impact on stickleback plate morph evolution in North Uist was unexpected. MacColl and Aucott’s (2014) conclusion that high predation rates drive the *loss* of protective plates, and spines is especially perplexing and we, like MacColl and Aucott (2014), are unable to formulate a coherent explanation for this finding. While our analysis of morphology failed to detect a role for predation, our behavioral data did indicate an effect, although this behavioral response could be a short-term learned response to attacks by predators, rather than an innate-evolved response. Without wishing to present an entire recapitulation of our original discussion from Spence et al. (2013), our working hypothesis is that the effects of dissolved calcium and predation interact. Hence, although sticklebacks may undergo selection pressure from predators for the elaboration of plates and spines, where calcium availability is limiting the capacity to respond to selection by predators through more extensive and robust plates and spines may be limited. Our data indicated that there is a threshold calcium concentration below which lateral plates and pelvic spines cannot evolve, irrespective of predation pressure. Thus, while we acknowledge the potential weakness of our predator data here (as we did in our original paper), for the reasons, we have discussed it is also probable that MacColl and Aucott’s (2014) estimates are similarly flawed. The challenge for both our groups is to obtain an alternative, independent estimate of trout abundance, that does not rely on angling records, and which includes the full range of sites on North Uist.

A final possibility is that neither dissolved calcium nor predation are the primary agents of selection on plate and spine morph. Several alternative suggestions for stickleback plate morph evolution have been proposed, including climate (Hagen and Moodie 1982), swimming regime (Baumgartner and Bell 1984), and buoyancy (Klepaker 1993). Further, selection can only act on individuals carrying the mutations necessary for plate, spine, and pelvic reductions, and the absence of genetic variance for these traits will inhibit morphological evolution, irrespective of selection regime (Klepaker and Østbye 2008; Klepaker et al. 2012). It is vital that we do not overlook these alternative mechanisms, or the role of multiple selective agents acting simultaneously, on patterns of morphological evolution in the design of future studies.
